# Benefits and Barriers to mHealth in Hypertension Care: Qualitative Study With German Health Care Professionals

**DOI:** 10.2196/52544

**Published:** 2025-03-10

**Authors:** Susann May, Felix Muehlensiepen, Eileen Wengemuth, Frances Seifert, Martin Heinze, Dunja Bruch, Sebastian Spethmann

**Affiliations:** 1 Brandenburg Medical School Theodor Fontane Center for Health Services Research, Faculty of Health Sciences Rüdersdorf Germany; 2 Brandenburg Medical School Theodor Fontane Department of Cardiovascular Surgery, Heart Center Brandenburg, Faculty of Health Sciences Bernau Germany; 3 Brandenburg Medical School, Immanuel Hospital Rüdersdorf University Clinic for Psychiatry and Psychotherapy Rüdersdorf Germany; 4 Deutsches Herzzentrum der Charité Department of Cardiology, Angiology and Intensive Care Medicine Berlin Germany; 5 corporate member of Freie Universität Berlin and Humboldt-Universität zu Berlin Charité – Universitätsmedizin Berlin Berlin Germany; 6 German Center for Cardiovascular Research (DZHK), Partner Site Berlin Berlin Germany

**Keywords:** hypertension, mHealth apps, digital health, physicians, nurses, HCP, qualitative interviews, health care professional, cardiologists, mHealth, Germany, general practitioners, blood pressure monitoring, qualitative study, qualitative content analysis

## Abstract

**Background:**

Digital health technologies, particularly mobile health (mHealth) apps and wearable devices, have emerged as crucial assets in the battle against hypertension. By enabling lifestyle modifications, facilitating home blood pressure monitoring, and promoting treatment adherence, these technologies have significantly enhanced hypertension treatment.

**Objective:**

This study aims to explore the perspectives of health care professionals (HCPs) regarding the perceived benefits and barriers associated with the integration of mHealth apps into routine hypertension care. Additionally, strategies for overcoming these barriers will be identified.

**Methods:**

Through qualitative analysis via semistructured interviews, general practitioners (n=10), cardiologists (n=14), and nurses (n=3) were purposefully selected between October 2022 and March 2023. Verbatim transcripts were analyzed using qualitative content analysis.

**Results:**

The results unveiled 3 overarching themes highlighting the benefits of mHealth apps in hypertension care from the perspective of HCPs. First, these technologies possess the potential to enhance patient safety by facilitating continuous monitoring and early detection of abnormalities. Second, they can empower patients, fostering autonomy in managing their health conditions, thereby promoting active participation in their care. Lastly, mHealth apps may provide valuable support to medical care by offering real-time data that aids in decision-making and treatment adjustments. Despite these benefits, the study identified several barriers hindering the seamless integration of mHealth apps into hypertension care. Challenges predominantly revolved around data management, communication contexts, daily routines, and system handling. HCPs underscored the necessity for structural and procedural modifications in their daily practices to effectively address these challenges.

**Conclusions:**

In conclusion, the effective usage of digital tools such as mHealth apps necessitates overcoming various obstacles. This entails meeting the information needs of both HCPs and patients, tackling interoperability issues to ensure seamless data exchange between different systems, clarifying uncertainties surrounding reimbursement policies, and establishing the specific clinical benefits of these technologies. Active engagement of users throughout the design and implementation phases is crucial for ensuring the usability and acceptance of mHealth apps. Moreover, enhancing knowledge accessibility through the provision of easily understandable information about mHealth apps is essential for eliminating barriers and fostering their widespread adoption in hypertension care.

**Trial Registration:**

German Clinical Trials Register DRKS00029761; https://drks.de/search/de/trial/DRKS00029761

**International Registered Report Identifier (IRRID):**

RR2-10.3389/fcvm.2022.1089968

## Introduction

Hypertension is a significant health problem affecting millions of people worldwide [1]. In Germany, too, hypertension is one of the most common diseases affecting a large proportion of the population. Prevention and effective treatment of hypertension are therefore crucial to prevent associated serious complications such as ischemic heart disease, strokes, and renal disease, and to improve the quality of life of those affected [2-4].

In recent years, the development of digital health technologies has experienced a considerable upswing, opening up promising perspectives for the prevention and management of hypertension [5]. Mobile health (mHealth) apps, wearables, and other digital tools enable patients to monitor their blood pressure levels, track lifestyle changes, and set health goals. These technological advances also offer health care professionals (HCPs) new opportunities to improve patient care and promote healthier lifestyles [6]. mHealth apps in particular have been shown to improve the treatment of hypertension [7].

In Germany, the following professional groups are primarily involved in the treatment of people with hypertension. General practitioners play a central role in the diagnosis, treatment, and long-term care of patients with hypertension. They are often the first point of contact for patients with hypertension and coordinate further care. Cardiologists specialize in the diagnosis and treatment of heart disease, including hypertension. They can treat complex cases of hypertension and offer specialized diagnosis and treatment options. Internists can also play a role in the care of patients with hypertension, especially if there are comorbidities or complex medical problems. Nursing professionals (nurses, nursing assistants) play a crucial role in the care of patients with hypertension. They help implement blood pressure control measures, educate patients, and help monitor symptoms and side effects [8]. In October 2020, physicians in Germany were allowed to prescribe digital therapeutics (DiGA [Digitale Gesundheitsanwendungen]) in standard care for the first time [9]. DiGAs can be prescribed by physicians for a 3-month use period and is fully reimbursed by the statutory insurance companies. To obtain approval, mHealth providers must demonstrate the safety, functionality, quality, data security, and a fundamental benefit in a clinical study to obtain DiGA status. Patients receive a written prescription for a DiGA based on respective indications (eg, depression) and have to send it to their insurance company.. At the time this study was conducted, there was no DiGA that is specific for the prevention of hypertension. In perspective, however, it is expected that mHealth apps for the prevention of hypertension will obtain DiGA status and will be made available to patients. In their recent study, Dahlhausen et al [10] pointed out the significant role of HCPs, specifically physicians, in promoting mHealth use among patients. In order to understand the implementation of mHealth into hypertension care, it is necessary to understand the perspectives of physicians, nurses, and medical assistants and their perception of benefits and challenges, as well as their information needs, in order to best integrate mHealth into hypertension care.

Nevertheless, a certain frustration is noticeable among physicians in Germany. Not least because there have been repeated technical problems in the past with the connection to the German telematics infrastructure (TI). The TI comprises a range of technical components that are relevant for operation in medical practices. These include the practice management system (Praxis Verwaltungssystem), which supports the organization and documentation of practice tasks, and the connector, a piece of hardware that enables access to the TI and communicates with the eHealth card terminal and the Praxis Verwaltungssystem via a secure network. The eHealth card terminal is used to use the electronic health card and to check practice ID cards. Other components include mobile card terminals, practice or institution ID cards, a virtual private network access service, and the electronic health professional card, which is required for various applications such as the electronic doctor's letter and prescriptions [11]. However, half of the physicians have to struggle with technical problems at least once a week, compared to 36% in 2020. Overall, frustration with the digitization process has increased [12].

While there is a wealth of research focusing on the patient perspective regarding digital health tools [13-15], there remains a lack of comprehensive evidence on how physicians perceive and integrate these technologies into their clinical practice. Despite the promising potential of digital health tools, the potential obstacles and barriers to widespread adoption of these technologies in the clinical setting have not been thoroughly explored. A deeper understanding of the structural and individual factors that influence the implementation and adoption of digital prevention approaches is imperative. Integrating digital prevention approaches into the health care system requires a clear delineation of the role and positioning of these technologies within the broader health care landscape. A thorough investigation of how digital health tools can be effectively integrated into the existing health care infrastructure and how they are perceived by the various stakeholders in the health care sector is crucial.

Addressing these research gaps can contribute significantly to a more nuanced understanding of how digital health and specifically mHealth apps can be optimally used for hypertension prevention. It can also identify potential barriers and pave the way for strategies to improve clinical adoption and seamless integration of these technologies. In this study, we explore the following research questions: Which benefits do HCPs see in mHealth apps for prevention? What are possible structural and individual barriers to the use of mHealth apps? How can the challenges be met?

## Methods

### Study Design

This paper reports on our exploration of the HCPs’ perspectives regarding mHealth apps in hypertension prevention. We conducted in-depth interviews with general practitioners, cardiologists, and nurses. This study is part of the DiPaH project [16], which examines structural and individual factors in different stakeholders that influence the use of digital preventive measures in patients with arterial hypertension in Germany.

### Ethical Considerations

This study was approved by the Ethics Committee of the Brandenburg Medical School Theodor Fontane (E-02-20220620). All experimental protocols were approved by a named institutional or licensing committee. All methods were carried out in accordance with relevant guidelines and regulations. Participants were informed verbally and in writing about the purpose, the procedure, the significance of the study, and the benefits and risks that may be associated with it and had the opportunity to ask questions. They were also informed that they had the right to withdraw their consent to participate in the study at any time, either verbally or in writing, without giving reasons. They were also informed that personal data would be recorded and stored, whereby the data would be pseudonymized, but that no data would be published that could be used to identify the person. For security reasons, the data received from the participants were always stored in a password-protected folder on a secure desktop computer. Written consent was obtained after participants were given the opportunity to ask questions. Patients were not involved in designing this study. Participants were offered €75.00 (approximately US $77.89) as an incentive for their participation in the study.

### Recruitment

Participants were selected using purposive sampling [17]. We included HCPs who were currently actively involved in the treatment of people with hypertension, such as general practitioners, cardiologists, and nurses. The participants were selected according to defined categories, aiming to generate a heterogeneous sample with the broadest variety of different perspectives [18]. The following categories were included: region of practice (rural vs urban), main area of practice (outpatient vs inpatient), and gender (male vs female). A further inclusion criterion was interest and willingness in participating in an interview. Participants were recruited both by systematic invitation and by snowball sampling. The following sources were used for recruitment (Textbox 1).

**Sources used for recruitment.**

- Social media accounts of the university (Brandenburg Medical School Theodor Fontane), such as Facebook, Instagram, and Twitter
- Association of Statutory Health Insurance Physicians Brandenburg (KVBB): newsletter
- General practitioners’ association Brandenburg: information study participation at events
- Personal contacts among colleagues (snowball sampling)
- Interested persons contacted the study team by telephone or e-mail, and it was checked in advance whether they could be included before an interview was conducted

### Data Collection

A preliminary semi-structured interview guide was drafted by a multiprofessional team (SM, FM, DB, and SSp). The semistructured interview guide consisted of open-ended questions that explored how the participants are attuned to digital applications and mHealth apps, what benefits and barriers they see in digital technologies for prevention, what information needs they have, and how these can be met. The focus was on hypertension apps with the following functions: educating about hypertension, monitoring blood pressure, and promoting adherence, although no specific apps were offered or recommended to participants. Sample interview questions included: Which mHealth apps in the context of hypertension treatment are used in your practice, or what experiences have you had with it? What benefits do you see in the use of mHealth apps? What barriers do you see in the use of mHealth apps? How well informed are you about mHealth apps, and where are knowledge deficits? In addition, sociodemographic data were collected, including profession, gender, age, number of inhabitants of the place of practice, duration of professional activity, and setting. To ensure clarity and relevance of the questions, we did a pilot test of the interview guide with 5 eligible participants recruited from clinics and outpatient physicians in the study’s catchment area (refer to Multimedia Appendix 1).

All interviews were conducted via telephone during October 2022 to March 2023. The interviews were recorded and transcribed verbatim in accordance with data protection guidelines. Data collection continued until no substantially new findings emerged and saturation of content was reached. Saturation of content is defined as code saturation, when no additional issues are identified, and meaning saturation, when no further dimensions, nuances, or insights of issues can be found [19]. We chose to conduct 27 interviews, as this number has proven to be adequate in previous studies to reach theoretical saturation in the exploration of digital approaches [20,21]. Field notes (a short summary of the interview) were taken following each interview to ensure better comprehensibility of the interview situation. The field notes themselves were not included in the analysis.

### Data Analysis

Qualitative analysis of the interviews was performed iteratively by the study team (SM and FM) based on Kuckartz’s structured qualitative content analysis [22] using MAXQDA Analytics Pro 2022, Release (version 22.1.0; Verbi GmbH). After transcription of the audio material, the analysis began with a screening of the interview texts, whereupon the interviews were coded. Relevant text passages from the interview material were coded according to a deductive-inductive procedure. Main categories and subcategories were formed inductively from the codes. Until a common understanding of all the emerging categories was achieved, consensus discussions were held continuously in the research group. Other categories were developed deductively based on the research questions and merged into the coding tree. By then, the process of gathering data had already concluded. Two researchers (SM and FM) separately applied the established category system to analyze the complete data set, ensuring that the process could be traced and replicated. The data was analyzed in German. To present the findings, significant excerpts from the transcriptions were chosen as representative quotes. These quotes were translated into English by native speakers and incorporated into the manuscript. The manuscript has been compiled in accordance with the COREQ (Consolidated Criteria for Reporting Qualitative Research; refer to Multimedia Appendix 2) [23].

## Results

In total, 27 interviews were conducted and analyzed until theoretical saturation was reached. The mean duration of the interviews was 42 (range 33-56) minutes. The mean age of the participants (N=27) was 50 (range 35-74) years. Most participants were female (14 female/13 male). A total of 14 cardiologists, 3 nurses, and 10 general practitioners participated. In total, 9 persons worked in the inpatient setting and 17 persons worked in the outpatient setting. One person worked in both settings. Four of the HCPs had already had experience with digital applications in daily practice or were also working with them. Detailed characteristics of study participants are shown in Table 1.

**Table 1:** Detailed characteristics of study participants.

Characteristics		Participants (N=27), n (%)
**Professional group**		
	General practitioner	10 (37)
	Cardiologist	14 (52)
	Nurse	3 (11)
**Duration of professional activity**		
	>10	9 (33)
	10-20	8 (30)
	21-30	7 (26)
	31-40	3 (11)
**Gender**		
	Female	14 (52)
	Male	13 (48)
**Age (years)**		
	>40	2 (7)
	40-50	11 (41)
	51-60	11 (41)
	<60	3 (11)
**Number of inhabitants**		
	>10,000	2 (7)
	10-100,000	11 (41)
	100,001-1 million	4 (15)
	<1 million	10 (37)
**Setting^a^**		
	Inpatient	10 (37)
	Outpatient	18 (67)

^a^One participant works in both the inpatient and outpatient settings.

### Themes

The analysis explored 7 key themes, categorized into benefits and barriers. Themes identified as benefits included (1) patient safety, (2) patient autonomy and support, and (3) support in medical care. Themes identified as barriers were organized by the following context: (4) handling of data, (5) in the context of communication, (6) in daily routines, and (7) in dealing with the systems.

### Benefits

**Figure 1:**
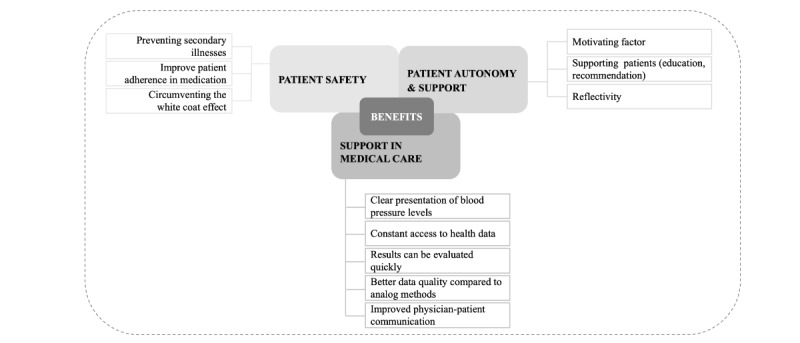
Coding tree of the benefits regarding mHealth apps in hypertension prevention. mHealth: mobile health.

Although few of the HCPs use or have used mHealth apps in their daily practice, they were able to mention benefits they expected to gain from using mHealth apps. In the context of benefits, 3 overarching themes could be identified: potential to increase patient safety, patient autonomy, and support in medical care (Figure 1 and Multimedia Appendix 3).

A different number of subcategories could be assigned to the 3 main categories. The following 3 subcategories were assigned to the “Patient safety” category: preventing secondary illnesses, improving patient adherence in medication, and circumventing the white coat effect. The following 3 subcategories were assigned to the category “Patient autonomy and support”: motivating factor, supporting patients, and reflectivity. The following 5 subcategories were categorized under the category “Support in medical care”: clear presentation of blood pressure levels, constant access to health data. Results can be evaluated quickly, better data quality compared to analog methods, and improved physician-patient communication.

#### Patient Safety

Respondents see the potential of mHealth apps primarily in the prevention of secondary diseases. In addition, participants see the potential of mHealth apps to improve patient adherence to treatment in terms of medication adherence, which in turn has an impact on patient safety.

The respondents attribute a better representation to the blood pressure levels measured at home and subsequently documented. The interviewees hope that through the use of mHealth apps, patients will consistently document their blood pressure and thus always have an overview of their blood pressure levels. This is because, in contrast to the blood pressure levels measured in the practice, they seem to correspond more closely to the reality of life. The “white coat effect” is thus circumvented.

#### Patient Autonomy and Support

An mHealth app can offer a variety of features that serve as a motivating factor for patients from the perspective of health care providers. Gamified elements, progress tracking, and reward systems can provide incentives for developing and maintaining health-promoting behaviors, strengthening patients' self-motivation, and fostering engagement in their health.

From the HCP’s perspective, an mHealth app can be a valuable addition to medical care by providing support to patients in several ways. First, the mHealth app can provide personalized advice based on the patient's individual needs and health goals. This can include dietary recommendations, lifestyle changes, or stress management tips. Second, mHealth apps can act as reminders, reminding patients to take their medication, which could improve adherence to therapy.

An mHealth app can also include reflectivity and help patients comprehend information about their blood pressure to their behaviors, which can support them reduce or avoid unfavorable behaviors.

#### Support in Medical Care

By using mHealth apps, the HCPs assume that blood pressure levels can be presented in a clear and understandable way. mHealth apps allow patients to access their levels on their smartphones or other digital devices. Patients can track the development of their blood pressure over time and identify possible trends or abnormalities so that they can seek medical help timely if necessary.

Patients have constant access to their current health data. This enables real-time tracking of their health status, even if they are not undergoing immediate medical treatment.

mHealth apps provide quick results that can be evaluated immediately. Physicians can thus make timely decisions about the treatment and care of their patients.

HCPs attribute better data quality to mHealth apps compared to analog methods.

Using mHealth apps, patients can send the data they collect in their daily environment directly to the medical facility before visiting the physician. This allows the physician to check the blood pressure levels in advance and prepare specifically for the conversation with the patient. The time saved helps to make the physician-patient conversation more efficient and targeted so that relevant questions and concerns of the patient can be better addressed.

The benefits initially included constant access to health data, which was particularly mentioned by participants in the outpatient sector. This group also appreciated the motivating factor that the apps could provide to support them in their health routines. Participants in large cities also preferred the app's clear display of their blood pressure readings, which helped them to better understand their health data. The time saved in doctor-patient communication was also mentioned as a benefit by the outpatient participants.

### Barriers

**Figure 2:**
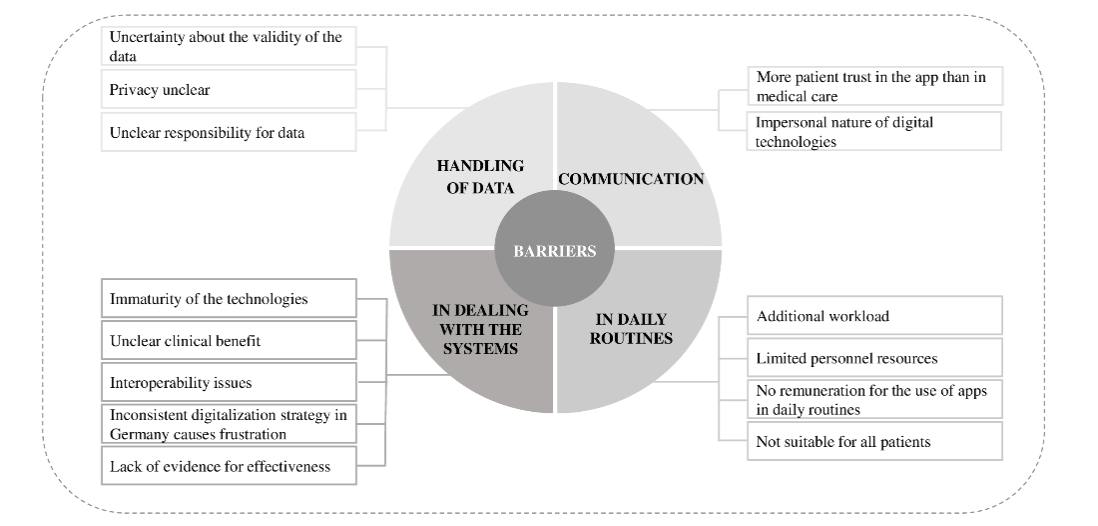
Coding tree of the barriers regarding mHealth apps in hypertension prevention. mHealth: mobile health.

We identified barriers in 4 different aspects of the HCPs work life: handling of data, in the context of communication, in daily routines, and in dealing with the systems (Figure 2 and Multimedia Appendix 4). A different number of subcategories could be assigned to the 4 main categories. The following 3 subcategories were assigned to the category “Handling of data”: uncertainty about the validity of the data, privacy unclear, and unclear responsibility for data. The following two subcategories were assigned to the category “in the context of communication”: more patient trust in the app than in medical care and the impersonal nature of digital approaches. The following 4 categories were assigned to the category “in daily routines”: additional workload, limited personnel resources, no remuneration for the use of apps in daily routines, and not suitable for all patients. The following 5 subcategories were assigned to the category “in dealing with the systems”: immaturity of the technologies, unclear clinical benefit, interoperability issues, and inconsistent digitalization strategy in Germany, which causes frustration and lack of evidence for effectiveness.

#### Handling of Data

One of the main barriers to the use of apps for blood pressure monitoring is uncertainty about the validity of the data collected. Concerns about the accuracy of data documented by patients can undermine HCPs’ confidence in the data and willingness to use such tools.

Another important concern related to the use of mHealth apps in the treatment of hypertension is privacy. Fear of data breaches or unauthorized access to patient information may limit the use of such tools.

Unclear responsibility for data presents another hurdle. Physicians and nurses feel uncertain about who is responsible for managing and interpreting the data they collect. The wealth of data generated by mHealth apps can be overwhelming, and the lack of clear structures and policies for appropriate use of the data can be challenging.

#### In the Context of Communication

The HCPs expect that a potential barrier to communication when using mHealth apps is the tendency of some patients to trust the mHealth app more than their medical care. This misconception could lead to patients relying solely on the mHealth app for solutions or seeing its advice as better than that of their physician. This could hinder communication with HCPs and lead to a lack of collaboration between patients and physicians.

Another barrier perceived by HCPs is the impersonal nature of mHealth apps. In contrast to direct interaction, the use of mHealth apps could lead to a lower intensity of exchange between patients and HCPs. In addition, mHealth apps may be perceived as less empathetic and less tailored to the individual needs of patients.

#### In Daily Routines

The HCPs apprehend that introducing mHealth apps in medical practice can initially lead to an additional workload for them. The introduction of new technologies requires training, the adaptation of workflows, and integration into existing IT systems, which can initially lead to time and resource constraints.

In many health care settings, resources are lacking in order to engage HCPs in the active use of mHealth apps in practice. Adoption and integration of new technologies requires time, training, and technical support, which can be challenging in an already strained health care system. Limited capacity impedes readiness to use mHealth apps for hypertension care.

At present, the use of mHealth apps is not sufficiently reimbursed in everyday health care in Germany. HCPs do not receive additional compensation or incentives for the use and deployment of digital tools in the care of hypertensive patients. This lack of compensation may cause some HCPs to be hesitant to use digital tools, as it may entail additional tasks without financial compensation.

Although mHealth apps offer many benefits, they may not be suitable for all patients. Older people or those with limited digital capabilities may have difficulty using digital applications. In addition, some patients, especially those with hypertension, may overmeasure their blood pressure, which can negatively impact their blood pressure levels. According to HCPs, digital tools for monitoring blood pressure may be less appropriate for such patients.

#### In Dealing With the Systems

A major barrier to the use of mHealth apps in hypertension care is the immaturity of the technologies. HCPs suspect that mHealth apps do not work reliably, have bugs, or do not have all the necessary features to meet the demands of medical practice. As a result, they may be reluctant to recommend digital technologies for fear that the systems will not exist at a later date.

Another barrier to the use of mHealth apps in hypertension care is the unclear actual utility and clinical effectiveness of such tools from the perspective of HCPs.

The diversity of mHealth apps and their interfaces is another barrier for HCPs. The incompatibility or lack of interoperability between systems can hinder smooth data exchange and integration.

The insufficient as well as inconsistent digitalization strategy in Germany is a source of frustration for HCPs. There is often a lack of a clear, long-term vision and of a structured approach to drive the integration of digital technologies in health care. The development and implementation of digital health solutions is often fragmented and inconsistent, leading to confusion, ineffective solutions, and a return to analog methods. These negative experiences are a significant barrier to the adoption of digital tools.

The actual effectiveness and long-term impact of mHealth apps in treating hypertension are poorly researched or demonstrated from the perspective of HCPs. The lack of evidence on the clinical effectiveness of such tools raises doubts and influences their willingness to use them.

#### Specific Aspects in Various Subgroups

Participants from the outpatient sector in particular pointed out interoperability problems that could hinder the smooth exchange of data. In addition, the unclear clinical benefits of the apps were not recognized by everyone, especially not by the participants from the outpatient sector. It was also noted from the outpatient sector that not all apps are suitable for every patient. Concerns about data protection were also expressed by participants in the outpatient sector, who were worried about the security of their personal health data. Finally, the lack of reimbursement for the use of health apps from the outpatient sector was cited as a further barrier. Women expressed concerns about the validity of the data collected by the apps.

#### Recommendations for Successful Integration of mHealth Apps Into the Health Care Landscape From HCPs’ Perspective

**Figure 3:**
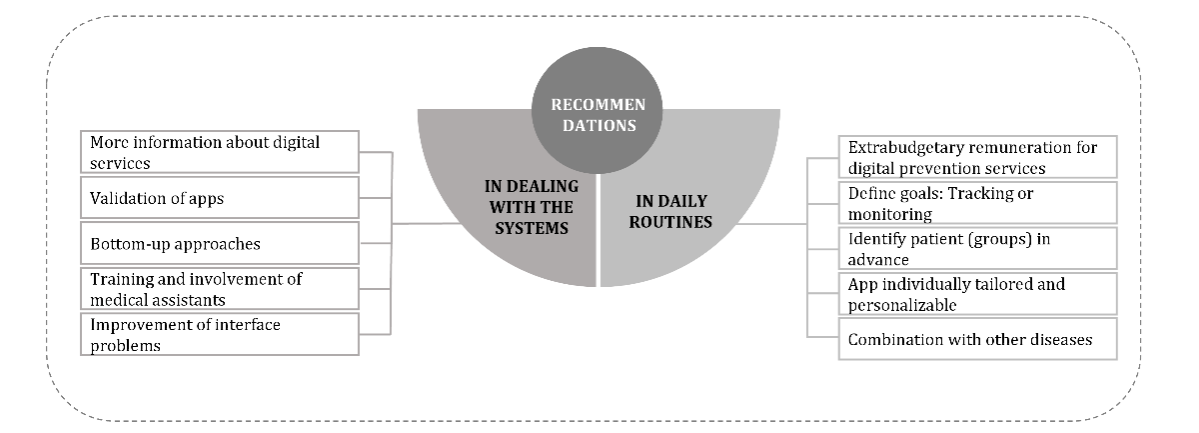
Coding tree of the recommendations for successful integration of mHealth apps into the health care landscape. mHealth: mobile health.

In the context of recommendations, 2 themes could be identified: in daily routines and in dealing with the systems (Figure 3 and Multimedia Appendix 5). A different number of subcategories could be assigned to the 2 main categories. The following 5 categories were assigned to the category “in daily routines”: extrabudgetary remuneration for digital prevention services, defining goals: tracking or monitoring, identifying patient (groups) in advance, app individually tailored and personalizable, and combination with other diseases. The following 5 subcategories were assigned to the category “in dealing with the systems”: more information about digital services, validation of apps, bottom-up approaches, training and involvement of medical assistants, and improvement of interface problems.

#### In Daily Routines

One of the fundamental challenges in integrating hypertension mHealth apps into routine medical practice is that the time spent using and counseling patients is not adequately compensated. To address this problem and increase HCPs' motivation to use digital prevention services, extrabudgetary reimbursement for the implementation and support of these apps in HCPs' practices should be considered. According to the participants, such remuneration would ensure that HCPs are appropriately compensated and thus create an incentive to increasingly integrate mHealth apps into their daily work.

It is crucial to define clear goals for the use of mHealth apps in hypertension prevention. HCPs should work with patients to determine which parameters or data should be collected by the mHealth app and which specific health goals are being pursued. The HCPs assume that a distinction can be made between tracking and monitoring approaches. While tracking helps patients to collect data on their blood pressure, lifestyle, and other relevant parameters themselves, monitoring enables the physician to regularly monitor and analyze the collected data. The clear definition of goals facilitates the use of the mHealth apps and ensures that the collected data can be used meaningfully and effectively.

The HCPs mentioned that not all patients benefit equally from mHealth apps. It is important to identify in advance which patients or patient groups could benefit most from a hypertension prevention mHealth app. Some patients may already be well informed and motivated to use digital health technologies on their own, while others may need more support and guidance. By identifying those patients who could best benefit from mHealth app use, clinicians can target their mHealth services more effectively and efficiently.

Another important improvement is that hypertension apps should be customized and personalizable. Each patient is unique, and the mHealth app should therefore be customizable to each individual's specific needs, health goals, and preferences. Personalization options could include selection of relevant health goals, medication reminders, or customization of the app interface to the user's personal preferences. By tailoring the app to each patient's individual needs, the user experience is likely to be enhanced and the likelihood of long-term use increased.

Hypertension often occurs in combination with other conditions, such as diabetes or obesity. To improve the care of multimorbid patients, mHealth apps for hypertension could be combined with other health applications to provide comprehensive and integrated care.

#### In Dealing With the Systems

It is important that HCPs have access to comprehensive and reliable information about the functionalities, efficacy, safety, and privacy policies of the various hypertension apps. A transparent and evidence-based presentation of this information can help to address concerns about the quality and reliability of the apps and stimulate physicians' interest in using these digital tools.

Careful validation of mHealth apps is essential to ensure that hypertension management apps actually deliver the promised benefits and provide medically robust data. HCPs need reliable information about the scientific evidence and clinical validity of the apps in order to effectively integrate them into their clinical practice.

To improve the adoption and integration of apps for hypertension, it is crucial to promote bottom-up approaches. This means including the user perspective, including physicians and medical staff, in the development and implementation of mHealth apps from the very beginning. Involving users in the development process makes it possible to adapt mHealth apps to the specific needs and requirements of physicians and ensure high usability.

Successful integration of hypertension apps requires not only training of physicians but also the involvement of other HCPs, especially medical assistants. Medical assistants play an important role in helping physicians use mHealth apps and interact with patients. Extensive training of medical assistants on how the mHealth apps work and are useful, as well as how to effectively use the data collected, can help ensure that the integration of the apps is seamless in the practice workflow.

Interoperability and compatibility between different mHealth apps and existing practice IT systems are critical. Often, physicians and medical assistants face the challenge that the interfaces between the mHealth apps and existing IT systems are not sufficiently optimized, which can lead to technical difficulties and data inconsistencies. Targeted improvement of the interface issues enables physicians to work smoothly with the mHealth apps and seamlessly integrate the captured data into their practice workflows.

## Discussion

### Principal Results

This qualitative study is the first to explore the factors that enable or hinder the use of mHealth apps for the prevention of hypertension from the perspective of general practitioners, cardiologists, and nurses in Germany. Until now, mHealth apps for the prevention of hypertension are only fragmentarily used in the care of hypertensive patients. From the perspective of the caregivers, the main potentials of mHealth apps lie in increasing patient safety, patient autonomy, and support in medical care. However, the presented results point to major challenges in data handling, patient-HCP communication, integration into standard care, and interoperability. Measures such as extra-budgetary remuneration for digital preventive services, setting targets through tracking or monitoring, identifying patient groups in advance, adapting and personalizing mHealth apps, and combining them with other diseases could, in the view of the HCPs, help to overcome the barriers. In dealing with the systems, the HCPs mention aspects such as more information about digital services, validation of mHealth apps, bottom-up approaches, training and involvement of medical assistants, and improvement of interface problems.

### Comparison With Prior Work

Our results are in line with earlier research in mHealth and beyond: physicians and nurses recognize the added values and positive potential of mHealth apps in supporting patients, fostering patient autonomy, and improving medication adherence [24,25]. The attributed benefits are supported by evidence: for instance, Li et al [26] report improvements in self-management behavior and medication adherence in adults using mHealth. Furthermore, self-monitoring of hypertension-related behaviors via smartphone apps combined with tailored advice has a modest but potentially clinically significant effect on blood pressure reduction [27].

Despite the advantages of mHealth, as of yet the barriers hinder their integration into the medical routine. One of the main concerns is the handling of data. Physicians frequently doubt the validity of data collected by patients and have concerns regarding data privacy and security. This can impede trust in the data and willingness to use such mHealth apps.

The mode of communication between physicians and patients can also be influenced by the use of mHealth apps. Some patients may tend to place more trust in the instructions provided by the mHealth app than in the recommendations of their medical caregivers. This could hinder communication with HCPs and impede collaboration between patients and physicians. However, studies have already demonstrated that there are contrary findings to the presumed concerns: findings of a systematic, narrative review show a generally positive influence of mHealth apps on physician-patient communication and relationships, which at times was correlated with better health outcomes [28]. And perhaps the use of apps can make visits more efficient, because blood pressure levels can be transmitted to the practice in advance, and conversations can be focused on essential aspects.

In the interviews, it is stated that mHealth consumes more resources and deteriorates patient communication. In rheumatology, digital health and especially mHealth are considered resource-saving. Digitization creates more time for patient interaction, which enhances the quality of care. If questions have already been answered beforehand, there may be extra time in the conversation with the patient to focus on essential aspects, which can positively impact the perceived quality of care [29]. Furthermore, it is emphasized in rheumatology care that this would allow more time for patient consultations. This is a contradiction. However, rheumatology is more digitized compared to hypertension care because of the greater shortage of personnel and disease burden.

Furthermore, apps can initially mean additional workload for HCPs. The introduction of new technologies requires training, adaptation of workflows, and integration into existing IT systems, which can lead to time and resource constraints. In addition, due to limited resources, many medical facilities may not be adequately prepared to actively integrate digital technologies into their practice. To be able to use digital technologies in their daily routine, physicians also need the appropriate digital skills [30]. In a recent measurement of physicians' professional digital health literacy, they scored 53.1 out of a possible 100 points [31]. For nurses, this figure is only slightly higher at 54.5 points. The most difficult task for physicians and nurses is “helping patients assess the trustworthiness of the digital health information they find,” followed by “helping patients find the digital health information that is relevant to them.” In the future, physicians and nurses will need support especially in this area so that the digital transformation in health care can be driven forward.

A lack of integration of digital tools into the reimbursement structure of the health care system in Germany is also a barrier to the use of such applications. Currently, physicians only receive remuneration for prescribing DiGAs, which does not translate to monitoring apps that have not obtained the DiGA status. There are ongoing discussions about which remuneration model is suitable for mHealth. Labinsky et al [32] reported that only 13% of patients that were prescribed a DiGA completed the whole program. This may also be due to a lack of incentives for physicians to ask if the apps are being used. Considering the low adherence with DiGA, performance-based compensation appears to be appropriate.

### Implications

Several recommendations for the integration of mHealth apps into medical practice can be derived from the results. It is important to provide HCPs with low-threshold access to comprehensive and reliable information about available mHealth apps to address their concerns about quality and privacy. Validation of mHealth apps for effectiveness and clinical validity is critical to convince physicians that these apps provide real value [33]. Bottom-up approaches or participatory study designs that include the user perspective, including physicians and medical assistants in the development process, can help ensure that apps are better tailored to the needs and requirements of medical practice and have a higher usability.

Involving HCPs, especially medical assistants, is also important as they play a key role in helping physicians use mHealth apps and interact with patients. Extensive training of medical staff on how the apps work and their benefits can help ensure that the integration of the apps is seamless in the practice workflow.

Our results underline the importance of staggered use of the mHealth app for effective blood pressure control in patients with hypertension. A high frequency of use at the beginning of treatment helps both patients and health care providers to quickly identify and implement individualized strategies to lower blood pressure. Once the target values have been stably achieved, the frequency of use can be gradually reduced so that the mHealth app can serve as a monitoring tool without unnecessarily increasing the time required by medical staff. This flexible handling could allow the mHealth app to be optimally integrated into everyday clinical practice while minimizing the burden on health care providers in terms of data management.

In addition, it is important to improve the interface issues between digital health apps and existing IT systems. Interoperability and compatibility between different digital apps and medical practice software systems are critical to ensure smooth integration and use of the apps. Targeted improvement of the interfaces can enable physicians to work smoothly with the apps and seamlessly integrate the collected data into their practice workflows.

The use of digital care services could be beneficial if the goal of use were addressed in advance in physician-patient communication. For example, use in the sense of self-tracking would not necessarily require medical intervention. Whereas the use in the sense of monitoring is associated with a control function, and physicians are actively involved in the process. Further studies are also needed to analyze the benefits and effectiveness of digital care services.

Based on the current findings and recommendations made by the HCPs, various stakeholders (eg, policy makers, health insurance companies, physicians’ associations, associations of the scientific medical societies, patient organizations, health care researchers, or app developers) in different fields of the health care system should take action to push the integration of mHealth app in daily practice (Textbox 2).

**Recommendations for the integration of mobile health (mHealth) apps into medical practice.**

**Structural conditions**

- Extrabudgetary remuneration for digital prevention services
- Improvement of interface problems
- Training and involvement of medical assistants
- More information about digital offers

**App development**

- Bottom-up approaches and user experience design
- App individually tailored and personalizable
- Combination with other diseases
- Validation of mHealth apps

**Use in patient care**

- Assessment of patients’ eligibility to use mHealth
- Identify patient (groups) in advance
- Define goals: tracking or monitoring

### Strength and Limitations

Our study is the first to investigate the benefits and barriers of using mHealth apps in preventing hypertension in Germany. We drew closely on the reality of care and the perspectives of the physicians and nurses. The qualitative interviews allowed for obtaining an in-depth understanding of the experiences with or without apps in preventing hypertension and expected advantages and disadvantages. However, there are certain limitations to our study. The results might be subject to a selection bias because perhaps people who were more interested in the topic took part in the study. Due to the sampling strategy, we may not have been able to reach everyone and did not record important aspects. A generalization of the results is therefore impossible. In addition, patients were not interviewed at this time.

The authors are aware that this general exploration can only be a first step given the breadth and diversity of the topic “hypertension mHealth apps.” Further studies must be conducted in the context of specific health care situations to build upon these findings. These limitations directly translate into opportunities for further research: We are planning to extend the study to a larger sample in Germany [16] and to validate the qualitative results with a questionnaire survey.

Further research should also focus on how to identify patients who may or may not be eligible for mHealth apps in hypertension care; how HCPs can be effectively informed about mHealth and digital health in general, as well as what reimbursement strategy will reinforce the effective use of mHealth in the care of chronic conditions.

### Conclusions

HCPs recognize the benefits of mHealth apps for patient safety or to support disease management. However, at present, best practices for implementing mHealth apps in preventing hypertension do not exist. In order to be able to use digital tools such as mHealth apps efficiently, the barriers, such as information needs, interoperability issues, uncertain remuneration, and vague clinical effectiveness, need to be overcome. Above all, users need to be involved in design and implementation processes. Furthermore, low knowledge needs to be eliminated by offering access to low-threshold information on mHealth apps.

## Appendix

Multimedia Appendix 1 Excerpt of the interview guide.

Multimedia Appendix 2 COREQ (Consolidated Criteria for Reporting Qualitative Research) checklist.

Multimedia Appendix 3 Coding tree with anchor quotes: benefits regarding mHealth apps in hypertension prevention. mHealth: mobile health.

Multimedia Appendix 4 Coding tree with anchor quotes: barriers regarding mHealth apps in hypertension prevention. mHealth: mobile health.

Multimedia Appendix 5 Coding tree with anchor quotes: recommendations for successful integration of mHealth apps into the health care landscape. mHealth: mobile health.

## Abbreviations

COREQ: Consolidated Criteria for Reporting Qualitative Research
DiGA: Digitale Gesundheitsanwendungen
HCP: health care professional
mHealth: mobile health
TI: telematics infrastructure

## Data Availability

All data generated or analyzed during this study are included in this published article (and its supplementary information files). For further questions regarding the reuse of data, please contact the corresponding author (susann.may@mhb-fontane.de).
