# Supplementary material for: Benefits and Barriers to mHealth in Hypertension Care: Qualitative Study With German Health Care Professionals
Source: JMIR Hum Factors. 2025 Mar 10;12:e52544. doi: 10.2196/52544 (PMC11933770; doi:10.2196/52544)
Supplement: Multimedia Appendix 1 [file humanfactors_v12i1e52544_app1.docx]

**Supplementary Material 1**

**Excerpt Interview Guide HPCs**

| Guiding question/ narrative impulse | Check aspects | Concretizing questions | Maintenance and control questions |
| --- | --- | --- | --- |
| What experiences have you already had with mHealth apps? | *Advantages - check aspects: Adherence to therapy, more information at an early stage, faster diagnosis, timely and effective therapy, more effective physician contacts, strengthening of the physician-patient relationship, networking of treating physicians*  *Disadvantages - check aspects: poor data protection, lack of or non-transparent guidelines on what happens to the data; imprecise measurements or malfunctions; faulty application; remuneration for consulting and support services provided by physicians in connection with health apps; uncertainties and concerns regarding reliability and ease of use* | What benefits do you see in the use of mHealth apps?  How do you rate the usability of mHealth apps by your patients?  How easy or difficult is it for you and your patients to use them in everyday life?  How well informed are you about mHealth apps and where are knowledge deficits?  What barriers do you see in the use of digital applications and mHealth apps?  What dangers or risks do you see when using mHealth apps?  What concerns do you have?  What could go wrong?  How could the challenges be met?  What can be done better?  And how can we do it better? | Can you tell more about this?  And then?  What was that like for you?  How do you see it?  Can you elaborate on that, please?  Could you give an example of that, please?  What do you mean specifically? |
