# Supplementary material for: Benefits and Barriers to mHealth in Hypertension Care: Qualitative Study With German Health Care Professionals
Source: JMIR Hum Factors. 2025 Mar 10;12:e52544. doi: 10.2196/52544 (PMC11933770; doi:10.2196/52544)
Supplement: Multimedia Appendix 3 [file humanfactors_v12i1e52544_app3.docx]

**Multimedia Appendix 3.** Coding tree with anchor quotes: benefits regarding mHealth apps in hypertension prevention

| Category | Subcategory | Anchor quote |
| --- | --- | --- |
| PATIENT SAFETY | Preventing secondary illnesses | *"Yes, I think so. Many patients go to emergency departments when their blood pressure is very high because they don't know how to help themselves. This could probably be intercepted. And, of course, secondary damages would also be reduced because if the patient walks around with high blood pressure for a long time, various types of organ damage are likely to occur."* (Participant K11, Position 37) |
|  | Improve patient adherence in medication | *"You can promote adherence a bit, ensuring that they really take their blood pressure medication or that they see that it's effective."* (Participant H1, Position 103) |
|  | Circumventing the white coat effect | *"And if they do it for three days, I can tell much more about someone's blood pressure from fifteen levels than if I determine the blood pressure in the practice once because the white coat effect shouldn't be underestimated."* (Participant H7, Position 5) |
| PATIENT AUTONOMY & SUPPORT | Motivating factor | *"It depends a bit on what the app does. If the app is only a pure documentation program and doesn't say much, then it's just a tool for recording. As soon as the app starts to regulate things, just like Weight Watchers, it doesn't matter who does it. Whether it's a human or an app. We all know that when such a device tells us to do more or that our blood pressure is not enough for today, or if the blood pressure is marked in red, then there is motivation for the patient to improve to make the app happy. That's motivating."* (Participant H6, Position 43) |
|  | Supporting patients (education, recommendation) | *"I think an app that supplements the patient with information, reminds them of regular tablet intake, or explains general measures or explains why each medication should be taken in a certain way will, in my opinion, take or could take work away from the doctor because they are better informed, and a better-informed patient is more compliant and ultimately healthier."* (Participant K3, Position 77) |
|  | Reflectivity | *"So the question is, what does such a digital health app for hypertension bring? It's a nice gimmick. It's about biofeedback. That means, how can I make it clear to people, a) you have high blood pressure, and b) when do you have it? Then it's exciting for them to see, so when I'm under work-related stress, when I'm stressed at school, suddenly I have high blood pressure, right?"* (Participant H7, Position 25) |
| SUPPORT IN MEDICAL CARE | Clear presentation of blood pressure levels | *"A good clear presentation, so that the programs really make better overviews than the 25 different variants that patients come up with. We have already received pie charts of blood pressure levels from patients that don't really help us."* (Participant H6, Position 57) |
|  | Constant access to health data | *"You always have your smartphone with you. You can provide this data to anyone, so individual groups of people. So, before I send this to someone with my notes, it is, of course, very quick to upload it somewhere or send it by email. That's perfect."* (Participant K8, Position 65) |
|  | Results can be evaluated quickly | *"It's good, and for me, it's quick to evaluate, which is wonderful."* (Participant H10, Position 25) |
|  | Better data quality compared to analog methods | *"The data quality is simply much better than when something is written down."* (Participant K2, Position 9) |
|  | Improved physician-patient communication | *"The physician-patient communication and relationship are improved because they receive much more data from the home environment and can discuss them."* (Participant K2, Position 43) |
