# Supplementary material for: Benefits and Barriers to mHealth in Hypertension Care: Qualitative Study With German Health Care Professionals
Source: JMIR Hum Factors. 2025 Mar 10;12:e52544. doi: 10.2196/52544 (PMC11933770; doi:10.2196/52544)
Supplement: Multimedia Appendix 4 [file humanfactors_v12i1e52544_app4.docx]

**Multimedia Appendix 4.** Coding tree with anchor quotes: barriers regarding mHealth apps in hypertension prevention

| Category | Subcategory | Anchor quote |
| --- | --- | --- |
| HANDLING OF DATA | Uncertainty about the validity of the data | *"And the dangerous thing is that I don't know how valid the data is [...]. There could be manipulation by the patient. Because the patient says, 'Oh, the device now shows 145, well, that's almost like 139.'"* (Participant K8, Position 43) |
|  | Privacy unclear | *"So, we also encounter data protection problems; we don't know if someone installs trojans on their device, it doesn't have to be intentional, but who knows what people do with their phones, and then I could end up with spyware on my PC."* (Participant H6, Position 29) |
|  | Unclear responsibility for data | *"So, all data that is transmitted needs to be reviewed, someone has to take responsibility for it and react if necessary. It is not entirely trivial in terms of effort, and the question always remains whether there will be immediate consequences."* (Participant K11, Position 21) |
| IN THE CONTEXT OF COMMUNICATION | More patient trust in the app than in medical care | *"Well, or rather, it becomes difficult to counter with anything medical because the patient says, 'Yes, but the app says this. It's made by people who must know what they're doing.' Then, 'Yes, that may be, but in your case, it's different. It's different for you.' It can be difficult with trust."* (Participant H3, Position 71) |
|  | Impersonal nature of digital approaches | *"And it's maybe a bit impersonal, that you can say, 'Okay, he should send us his app overview once a quarter.' Then I get it electronically, and there's a name attached, and then they get a thumbs-up or thumbs-down in response or something like that."* (Participant H3, Position 63) |
| IN DAILY ROUTINES | Additional workload | *"I believe it fails simply because I also have little time, honestly, to deal with it. It's always more effort."* (Participant K3, Position 9) |
|  | Limited personnel resources | *"Even if hypertension is a very, very important issue, it's one of many, many issues, and everything comes together in general practice. Yes, there are limited capacities, personnel-wise, resource-wise, and if you, as we have found, have a practice that doesn't even have an email address [...] then this thinking - it's not meant to be mean, but - this thinking doesn't reach the point where there is something else, right, where these possibilities exist. If I don't understand or don't know or don't want to deal with it, for whatever reason, then I don't consider it in my daily work, using it."* (Participant H3, Position 27) |
|  | No remuneration for the use of apps in daily routines | *"It's unrealistic* [the use of m-health apps]. *That's just it, and as long as it's not compensated, nothing will happen, certainly not."* (Participant K7, Position 81) |
|  | Not suitable for all patients | *"And then there are also patients who constantly measure their blood pressure all day long, and for them, their entire life revolves around their condition. And that is a huge danger, especially with hypertension because the condition can trigger itself. So, we have to think carefully about which patients are suitable for which services and what the service is aiming for."* (Participant K14, Position 7) |
| IN DEALING WITH THE SYSTEMS | Immaturity of the technologies | *"It's of no use if I start something, and it will be scrapped in two years, and I have to tell all patients, 'Sorry.' That would be the worst for us. It's not yet mature in Germany."* (Participant K5, Position 31) |
|  | Unclear clinical benefit | *"Well, especially in hypertension, I wouldn't know its effect. Digital prevention, regarding atrial fibrillation, monitoring via smartwatches, I see the benefit there. But especially in hypertension, I basically see no benefit for my patients."* (Participant H5, Position 11) |
|  | Interoperability issues | *"The disadvantage is that it is not compatible with practice systems. Mostly."* (Participant K7, Position 85) |
|  | Inconsistent digitalization strategy in Germany causes frustration | *"Ultimately, both in hospitals and in practices, I have had the experience that the digital things, which are also offered or forced by the legislature, often did not work well. And, in fact, led to more work because the analog way was continued, and in the end, a double structure was created, and possibly also incurred costs from the providers. This frustrates me in Germany immensely."* (Participant K1, Position 5) |
|  | Lack of evidence for effectiveness | *"Well, and I mean, it's like, if I prescribe Ramipril, then I know that medication, I know its effects, side effects, I've used it for a long time, I know it. With these apps, which can be quite expensive, costing maybe 500 euros or so, I would like to know beforehand how effective it is when I prescribe it."* (Participant H10, Position 7) |
