# Supplementary material for: Benefits and Barriers to mHealth in Hypertension Care: Qualitative Study With German Health Care Professionals
Source: JMIR Hum Factors. 2025 Mar 10;12:e52544. doi: 10.2196/52544 (PMC11933770; doi:10.2196/52544)
Supplement: Multimedia Appendix 5 [file humanfactors_v12i1e52544_app5.docx]

**Multimedia Appendix 5.** Coding tree with anchor quotes: recommendations for successful integration of mHealth apps into the healthcare landscape

| Category | Subcategory | Anchor quote |
| --- | --- | --- |
| IN DAILY ROUTINES | Extrabudgetary remuneration for digital prevention services | *"So, the ideal approach would be, if I receive special compensation when I have my patients as healthy as possible, and on that path, it is up to me to use such methods, and I can take advantage of the complete range of possibilities available. If I say, okay, digital offerings, healthcare services, preventive measures are a good way, and I can use them effectively to make my patients even healthier or maintain their health, and then get compensated for it, then everything is right.”* (Participant H3, Position 55). |
|  | Define goals: Tracking or monitoring | *“From the beginning, it must be clear what the doctor and the patient aim to achieve together. The mHealth app contributes to evaluating and reviewing the achievement of goals. Is it only for the patient, or is the doctor involved as well? Then you can look at patient group X, but you probably already have those who don't have it, and the patient group that does.”* (Participant K14, Position 81) |
|  | Identify patient (groups) in advance | *"For a certain patient group, it is quite useful; you can manage them even better, but unfortunately, it's not suitable for others. And you have to identify that beforehand.”* (Participant H7, Position 33) |
|  | App individually tailored and personalizable | *“Yes, I think the mHealth app should have the possibility to be individually tailored, perhaps medically indicated, triggered by the doctor, programmed.”* (Participant K14, Position 77-79) |
|  | Combination with other diseases | *“They need an app that is more comprehensive. We have to address everything* [other diseases]*.”* (Participant K5, Position 51) |
| IN DEALING WITH THE SYSTEMS | More information about digital services | *„But for hypertension, I'm not aware of active promotion. I think we should be informed more about these digital services.”* (Participant H1, Position 41) |
|  | Validation of apps | *“On the other hand, we won't be able to get around creating good mHealth apps. MHealth apps that have been medically validated and have the appropriate claim” .*(Participant K14, Position 133) |
|  | Bottom-up approaches | *“And they should involve the users. With all these applications, you regularly get the feeling that they haven't spoken to a single family doctor who will ultimately use it. Otherwise, no one would have come up with the idea of developing an electronic prescription that only works through an app. If they were to sit at our counter for a day to see who comes and how often we still write appointment slips by hand.”* (Participant H6, Position 85) |
|  | Training and involvement of medical assistants | *“Of course, you can also imagine that something like this doesn't necessarily have to be dealt with by the doctor himself, but it can also be handled by qualified medical assistants, provisional assistants, and so on.”* (Participant K5, Position 21) |
|  | Improvement of interface problems | *„And it must be automated that this data goes into the practice software and that it works with interfaces. Otherwise, it's a stillborn child.“* (Participant K7, Position 85) |
